# Mapping of research on maternal health interventions in low- and middle-income countries: a review of 2292 publications between 2000 and 2012

**DOI:** 10.1186/s12992-016-0189-1

**Published:** 2016-09-06

**Authors:** Matthew Chersich, Duane Blaauw, Mari Dumbaugh, Loveday Penn-Kekana, Siphiwe Thwala, Leon Bijlmakers, Emily Vargas, Elinor Kern, Josephine Kavanagh, Ashar Dhana, Francisco Becerra-Posada, Langelihle Mlotshwa, Victor Becerril-Montekio, Priya Mannava, Stanley Luchters, Minh Duc Pham, Anayda Gerarda Portela, Helen Rees

**Affiliations:** 1 Wits Reproductive Health and HIV Institute, Faculty of Health Sciences, University of Witwatersrand, Johannesburg, South Africa; 2 Centre for Health Policy and MRC Health Policy Research Group, Faculty of Health Sciences, University of Witwatersrand, Johannesburg, South Africa; 3 University of Basel, Basel, Switzerland; 4 Department of Epidemiology and Public Health, Society, Gender and Health Unit, Swiss Tropical and Public Health Institute, Basel, Switzerland; 5 Department of Infectious Disease Epidemiology, London School of Hygiene and Tropical Medicine, London, UK; 6 Radboud University Medical Center, Radboud Institute for Health Sciences (RIHS), Nijmegen, The Netherlands; 7 Innovation in Public Health Department, National Institute of Health, Bogotá D.C, Colombia; 8 National Institute of Public Health (Instituto Nacional de Salud Pública)/Centre for Health Systems Research, Cuernavaca, Mexico; 9 Pan American Health Organization, Washington D.C, USA; 10 Centre for International Health, Burnet Institute, Melbourne, VIC Australia; 11 Department of Epidemiology and Preventative Medicine, Monash University, Melbourne, Australia; 12 Department of Maternal, Newborn, Child and Adolescent Health, World Health Organization, Geneva, Switzerland; 13 London School of Hygiene and Tropical Medicine, London, UK

**Keywords:** Maternal health, Maternal mortality, Low- and middle-income countries, Health systems, Research governance, Health promotion, Systematic mapping

## Abstract

**Background:**

Progress in achieving maternal health goals and the rates of reductions in deaths from individual conditions have varied over time and across countries. Assessing whether research priorities in maternal health align with the main causes of mortality, and those factors responsible for inequitable health outcomes, such as health system performance, may help direct future research. The study thus investigated whether the research done in low- and middle-income countries (LMICs) matched the principal causes of maternal deaths in these settings.

**Methods:**

Systematic mapping was done of maternal health interventional research in LMICs from 2000 to 2012. Articles were included on health systems strengthening, health promotion; and on five tracer conditions (haemorrhage, hypertension, malaria, HIV and other sexually transmitted infections (STIs)). Following review of 35,078 titles and abstracts in duplicate, data were extracted from 2292 full-text publications.

**Results:**

Over time, the number of publications rose several-fold, especially in 2004–2007, and the range of methods used broadened considerably. More than half the studies were done in sub-Saharan Africa (55.4 %), mostly addressing HIV and malaria. This region had low numbers of publications per hypertension and haemorrhage deaths, though South Asia had even fewer. The proportion of studies set in East Asia Pacific dropped steadily over the period, and in Latin America from 2008 to 2012. By 2008–2012, 39.1 % of articles included health systems components and 30.2 % health promotion. Only 5.4 % of studies assessed maternal STI interventions, diminishing with time. More than a third of haemorrhage research included health systems or health promotion components, double that of HIV research.

**Conclusion:**

Several mismatches were noted between research publications, and the burden and causes of maternal deaths. This is especially true for South Asia; haemorrhage and hypertension in sub-Saharan Africa; and for STIs worldwide. The large rise in research outputs and range of methods employed indicates a major expansion in the number of researchers and their skills. This bodes well for maternal health if variations in research priorities across settings and topics are corrected.

**Electronic supplementary material:**

The online version of this article (doi:10.1186/s12992-016-0189-1) contains supplementary material, which is available to authorized users.

## Background

Despite major advances in improving maternal health over the past 15 years, progress in many countries was too slow to achieve the targets set for Millennium Development Goal 5 (MDG-5) [1, 2]. Progress lagged behind, especially in sub-Saharan Africa and Southern Asia, while some areas have made tremendous advances towards their targets, such as Eastern Asia, Caucasus and Central Asia [3]. Most importantly, however, still one in four babies worldwide are born without skilled care [2]. Inequities in maternal health also remain particularly concerning and may even have risen over the MDG period [4]. The estimated lifetime risk of maternal mortality in high-income countries (HICs) is 1 in 3300, in comparison with 1 in 41 in low-income countries (LICs) [1]. In low- and middle-income countries (LMICs), women in poorer income quintiles or living in rural areas experience several-fold higher rates of maternal death than their richer and urban-dwelling counterparts [2, 4, 5]. Of note, while marked progress has been made in decreasing maternal deaths from HIV, considerably smaller reductions have occurred in deaths from haemorrhage and hypertension [6]. These inequities and differential progress between maternal health conditions question whether the maternal health strategies and the current research agenda are aligned with the needs of pregnant women in LMICs, especially among more vulnerable groups.

While many policy makers hold that the strategies for securing safe motherhood are now well described [7], considerable weaknesses remain in the evidence base required to secure further improvements, and approaches may well need to be country and region specific. Also, it may be argued, for instance, that the evidence generated to date has largely focused on clinical interventions to reduce morbidity and mortality, with relatively less attention given to the factors that determine their effectiveness and uptake. These are principally related to the functionality of health systems and health promotion [8–10]. The latter influence the reach, accessibility and acceptability of clinical interventions.

Using content analysis of a systematic mapping of literature, this study describes the characteristics of interventional research on maternal health in LMICs between 2000 and 2012. Given the importance of health systems and health promotion research, the study especially aims to identify patterns in research on these topics and to contrast these with trends in research on individual clinical conditions, such as HIV and hypertension. Also, comparing the number of studies and of deaths from individual clinical conditions can help determine whether the research done matches the key causes of maternal deaths. Similarly, contrasting the number of studies in different countries, and trajectories over time, allows for inferences to be made about the priority given to maternal health interventional research in different settings. Overall, the study could assist in identifying key gaps in research activities, and how priorities might be redirected.

## Methods

### Identification of literature and database management

The paper summarises the findings of a large-scale systematic mapping of all maternal health interventional research in LMICs published between 01/01/2000 and 31/08/2012 [11]. The systematic mapping, using full text publications, covers a broad body of literature on maternal health, and differs from a classic systematic review that addresses a single, narrowly-defined research question [12]. It uses established methods for producing systematic maps, including those with an emphasis on health equity [13, 14]. In the mapping we identify and describe all papers published on this broad topic, but do not assess the quality of the included research.

A sensitive search strategy was developed for the mapping, using both controlled vocabulary and free-text terms to identify studies in Medline (PubMED). The search strategy was then adapted for searching other electronic sources, namely CINAHL, Embase, LILACS, PopLINE, PsycINFO and Web of Knowledge (Additional file 1). Methods used in the search strategy are described elsewhere in more detail [15].

We included studies in LMICs that targeted women in pregnancy, during childbirth or within 2 years postpartum, or men within maternal health services. Studies had to include health systems, health promotion or community-based interventions; or interventions on one of five clinical tracer conditions: haemorrhage, hypertension, HIV, sexually transmitted infections other than HIV (STIs), or malaria. The first two of these tracers were selected as they constitute the leading causes of direct maternal deaths [6, 16, 17], while HIV and malaria are the principal causes of indirect maternal deaths in some LMICs [1, 6]. STIs other than HIV remain a key, but neglected, cause of maternal and newborn morbidity and mortality [18, 19]. General health system interventions were included only if they reported outcomes in a maternal health population. We excluded articles related to infertility. Descriptions of population coverage of routine services were also excluded, given difficulties in standardising data extraction from these very diverse studies over a large study team (15 reviewers across 8 countries). All study designs were included, aside from narrative reviews and policy discussion papers. Studies could be in Arabic, English, French, Portuguese or Spanish.

Management of the database, screening for eligibility and data extraction were done using online systematic review software (EPPI-Reviewer 4; http://eppi.ioe.ac.uk/cms/). Data extraction codes were piloted and then refined. Reviewers received training on screening articles for eligibility and data extraction. Of the 45,959 articles initially uploaded, 10,881 were duplicate items (23.7 %; Flow Chart). The titles and abstracts of the remaining records (35,078) were screened independently by two reviewers. Differences between reviewers were resolved by a third, more senior reviewer. A total of 18,386 articles were identified on maternal health, of which 16,094 were excluded. The most common reason for exclusion was the absence of an intervention or study outcome (10,536). Almost 4500 studies of clinical interventions other than the tracer conditions were identified and excluded (4450). Of 4175 full text papers reviewed, 2292 were included in the final mapping (54.9 %); data were then extracted from these papers.

### Study variables

The list of LMICs and their respective economic category (low-income, low-middle income and upper-middle income) was based on the World Bank classification [20]. Each study was categorised as being a systematic review, effectiveness study (non-experimental quantitative assessment of effectiveness, such as time series and cohort studies), randomised controlled trial (RCT), qualitative study, modelling study or mixed methods research. Systematic reviews and modelling studies were not classified as pertaining to research activities in a particular country, unless it specifically focused on a country.

Interventions were classified as targeting one or more of pregnancy, intrapartum or the postpartum, and a specific population, such as women, men, traditional birth attendants (TBAs), and programme managers. Attention given to health inequities was assessed by identifying the proportion of articles that determined intervention outcomes across different categories of social differentiation, as defined by the mnemonic PROGRESS-Plus: **P**lace of Residence, **R**ace/Ethnicity, **O**ccupation, **G**ender, **R**eligion, **E**ducation, **S**ocioeconomic status and **S**ocial Capital, Age and Disability [21]. As per the WHO framework for health systems [22], health systems interventions were defined as actions undertaken to improve the functioning of one or more of the five WHO Health Systems Building Blocks, or to enhance access, coverage, efficiency, or quality of maternal health services. Health promotion interventions-implemented either within communities or at health facilities-encompassed activities targeting, for example, TBAs, men, transport and demand-side financing [23]. Data on the journal’s Impact Factor were downloaded from Thomson Reuters [24] and the total number of health publications in 2000–2011 was extracted from Rottingen JA et al. [25].

### Data analysis

Data checks were performed in the EPPI-Reviewer software and in Stata 13 (StataCorp LP, College Station, TX, USA); the latter was also used for analysis. Characteristics of research on health systems and health promotion, and the five clinical tracers were compared across settings, time, study design and populations targeted. We also examined the proportion of studies on each of the clinical tracers that included a health systems or health promotion component. The distribution of Impact Factor of the journal and proportion of studies that were RCTs were used as a proxy for the quality of the research done. Only countries with five or more publications were included in cross-country comparisons; those with fewer papers were grouped together. To identify changes over time, publication rates were calculated for three time periods (2000–2003, 2004–2007 and 2008–2012). For comparison, the total number of papers in the last time period (01/2008-08/2012) was multiplied by a factor of 0 · 86, as this period was 4 · 67 years, while the other periods were 4 years each.

To assess alignment between the research outputs and the burden of disease from different conditions, we compared the number of articles with the estimated number of women dying from the condition in different settings [6]. Total numbers of papers in the review from each country and geographical region was contrasted with the burden of maternal deaths in that country or region [6]. We compared the number of publications on HIV interventions in different countries with the number of HIV-infected pregnant women in 2012 [26]. The numbers of papers per country and per region were also compared with the average GDP (2000–2012) [27]. Finally, to assess the priority given to maternal health research in each country, we determined the proportion of all health publications in 2000–2011 that described a maternal health intervention.

Chi square tests were used to detect associations between categorical variables, and the Chi square test for trend to identify changes over time. The Mann-Whitney U test identified associations between Impact Factor and other study variables. Multiple responses were possible for many variables, given that some studies involved more than one country, or addressed several populations and topics. The sum of percentages for these variables may thus exceed 100 %.

## Results

### Characteristics of the studies (Flow chart and Table **1**)

**Table 1:** Topics addressed in maternal health interventional research between 2000 and 2012: setting, design and intervention type (column percentages)

	Health systems (830) Col. %	Health promotion (626) Col. %	Clinical intervention (1533) Col. %	Type of clinical intervention					Total % (column)	Total N
				APH/PPH (202) Col. %	Hypertension (232) Col. %	HIV (792) Col. %	STIs (122) Col. %	Malaria (283) Col. %		
Time period										
2000–2003	**17.8**	**15.5**	**17.6**	19.2	19.2	17.1	**27.4**	14.6	17.7	370
2004–2007	**32.6**	**33.4**	**38.8**	37.9	37.6	38.8	**44.6**	37.2	37.0	773
2008–2012	**49.6**	**51.1**	**43.6**	42.9	43.2	44.1	**28.0**	48.2	45.3	947
Economic region^a^										
LIC	**42.2**	**45.8**	37.3	**23.3**	**18.5**	**40.4**	*28.9*	**55**	37.2	724
LMIC	33.3	31.7	**30.5**	**53.4**	*25.8*	**24**	33	36.5	32.1	624
UMIC	**29**	**25.8**	*36.8*	*29.4*	**59.6**	**40.4**	**47.4**	**10.8**	35.4	688
Geographic region^a^										
East Asia Pacific	12.6	11.9	**10.4**	*16*	*7.3*	**8.8**	**19.6**	9.2	11.6	226
Europe, Central Asia	2.5	2.4	3	**6.1**	**9.9**	**1.7**	4.1	**0**	3.2	63
Latin America, Caribbean	17.1	16.3	**15.1**	16	**29.1**	15.4	**26.8**	**0.8**	16.7	325
Middle East, North Africa	2.6	3	2.4	**10.4**	**7.9**	**0**	3.1	**0**	2.8	54
South Asia	**19.7**	**23.7**	**8.7**	**25.2**	**21.2**	**4.5**	9.3	**2.8**	13.7	267
Sub-Saharan Africa	**48.9**	**45.7**	**63.6**	**39.3**	**31.1**	**70.8**	**43.3**	**88**	55.4	1080
Multi-country study										
Multi-country study	7.1	5.9	7.7	9.9	7.3	8.5	6.6	5.3	7.2	166
Study design										
Systematic review	**6.5**	**9.7**	**10.6**	**11.4**	**30.6**	**5.7**	**9.8**	**9.2**	10.8	247
Effectiveness research	**69.9**	**61.5**	**60.5**	**57.4**	**49.1**	**65.9**	**62.3**	**55.8**	61.4	1408
RCT	**5.2**	**11.2**	**15.8**	**21.8**	**15.1**	**12.8**	**9.8**	**21.2**	13.1	301
Qualitative study	**7.1**	**7.7**	**4**	**1.5**	**0.9**	**5.9**	**1.6**	**2.5**	5.3	121
Modelling	**7.5**	**4.5**	**6.7**	**5.9**	**3.9**	**6.9**	**14.8**	**7.8**	6.5	148
Mixed methods research	**3.9**	**5.4**	**2.5**	**2**	**0.4**	**2.8**	**1.6**	**3.5**	2.9	67
Impact Factor										
None	**61.3**	**63.1**	**57.6**	**61.4**	**47.4**	**59**	**47.5**	**66.4**	58.8	1348
0–1	**18.3**	**15.8**	**15.5**	**24.8**	**31**	**11.4**	**17.2**	**7.4**	16.4	375
2–4	**15.2**	**15.2**	**18**	**8.4**	**14.7**	**18.4**	**32**	**19.1**	16.8	385
≥ 5	**5.2**	**5.9**	**8.9**	*5.4*	**6.9**	**11.2**	*3.3*	**7.1**	8	184
Study examines inequalities										
Yes	**14.3**	**20.9**	**5.9**	9.9	**2.2**	**4.5**	*4.1*	10.2	9	206
Period targeted^a^										
Pregnancy	**62.8**	70.6	**76.7**	**17.3**	**85.8**	**80.2**	**95.9**	**96.5**	70.4	1613
Childbirth	**65.5**	**59.6**	**41.2**	**89.1**	44.8	**44.7**	**22.1**	**9.5**	49.9	1144
Postpartum	**40.7**	**41.2**	**27.8**	32.7	**13.8**	**39**	**18**	**12.7**	32	734
Intervention recipient^a^										
Women	**56.3**	78	**88.6**	75.7	**85.3**	**89.9**	77.9	**90.8**	79.1	1812
Males	**4.1**	**10.5**	2.7	*1*	**0**	**4.5**	3.3	**0.4**	3.1	72
Family	**4.8**	**7.8**	**1.9**	3	**0**	**1.5**	0.8	3.9	2.7	63
Community	**8.6**	**16.9**	**2.3**	5.4	**0.9**	**1.5**	**0**	3.9	4.6	106
TBA	**8.4**	**13.3**	**1.7**	**9.4**	*1.7*	**0.1**	**0**	2.5	3.9	89
Medical doctor	**15.7**	**8.5**	**3.7**	**13.9**	8.2	**2.7**	*2.5*	**1.4**	6.7	154
HCW other than doctor	**25.5**	**14.1**	**5.7**	**19.8**	*7.3*	**4.5**	7.4	**2.8**	11	251
Community health worker	**8**	**10.2**	**1.6**	4	**0.9**	**1.1**	0.8	2.1	3.4	79
Managers and planners	**33.5**	*18.4*	**9.7**	13.9	**11.2**	**8.6**	**23.8**	**10.2**	15.9	365
Includes health system component										
Yes	100	**64.5**	**14.9**	**29.7**	**14.7**	**12.8**	**23**	**17**	36.2	830
Includes health promotion component										
Yes	**48.7**	100	**13.9**	22.8	**9.5**	**12.1**	**12.3**	**20.8**	27.3	626
Outcomes reported^a^										
Maternal health	**42**	**44.6**	**62.5**	**77.2**	**84.9**	**49.4**	62.3	**70**	56.2	1287
Child health	**24.1**	**29.7**	**45.5**	**13.9**	**53.9**	**49.5**	38.5	**49.8**	38.9	891
Service delivery	**48.7**	**53**	**25.2**	**16.8**	**4.7**	33.2	**23**	**24**	31.4	719
Health economics	**14.2**	**12.8**	**6.2**	6.9	*4.7*	**4.8**	**18**	8.8	7.9	181
Other	**23.3**	**19**	**8.9**	12.9	**8.6**	**9**	*8.2*	**7.4**	14.1	324

Figures in bold *P* < 0.05. Figures in italics *P* = 0.05-0.1. APH/PPH antepartum and postpartum haemorrhage. STIs sexually transmitted infections. ^a^Multiple-response categories. Clinical interventions encompass studies on haemorrhage, hypertension, HIV, sexually transmitted infections other than HIV, or malaria. Effectiveness research excludes RCTs

Of the 18,386 articles identified on maternal health, about two thirds were descriptive in nature and they were therefore excluded (63.3 %, 11644; Flow chart). The remaining 36.6 % (6742) were interventional studies, of which 2292 were included (34.0 %). Almost 80 % of the 6742 interventional studies (79.0 %; 5329) addressed a single clinical condition; 9.7 % of the 6742 studies covered clinical conditions that also included a health system or health promotion component (654), and 11.3 % addressed only a health system or health promotion intervention (759).

**Fig. 1:**
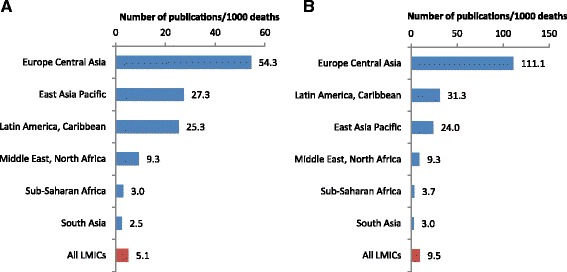
Number of publications per 1000 maternal deaths from haemorrhage (**a**) and hypertension (**b**), by region

HIV research constituted around half of the 1533 articles on the five tracer conditions (51.7 %, 792; multiple-response question: Table 1), and together with malaria made up 69.1 % of these studies (1059/1533). Of the remaining articles on the tracers, about a quarter covered hypertension or haemorrhage, and 5.4 % were on STIs. The number of publications per 1000 maternal deaths from hypertension (9.5 %) was almost double that of haemorrhage (5.1 %; Fig. 1). More than a third of articles on haemorrhage included a health systems or health promotion component, as did a quarter of malaria studies. By comparison, only 15 % of HIV studies did so, the lowest of the clinical topics reviewed. HIV, hypertension and STI research were least likely to examine inequalities.

Overall, aside from haemorrhage studies, the other clinical interventions overwhelmingly targeted pregnancy, while systems and health promotion studies paid relatively more attention to childbirth and the postpartum period (Table 1). Only around five percent of all interventions included community services as part of the intervention studied (4.6 %), 3.9 % of articles investigated interventions among TBAs and a negligible proportion involved men (3.1 %). These kinds of groups were even less frequently the subjects of studies of the tracer conditions. Intervention recipients for studies on haemorrhage commonly included doctors (13.9 %), other cadres of health workers (19.8 %) and community health workers (4.0 %). The corresponding figures for hypertension research were much lower: 8.2, 7.3 and 0.9 %, respectively. About half of health systems and health promotion research reported service delivery outcomes (such as levels of service uptake), while clinical tracer studies assessed mostly maternal (62.5 %) and child outcomes (45.5 %). Maternal and child outcomes were assessed in equal measure in HIV research, unchanged over time. By contrast, haemorrhage studies presented maternal outcomes 5.6 times more commonly than child outcomes, and the corresponding figures were 1.6 fold for hypertension and 1.4 fold for malaria. The proportion of studies reporting health economics analyses was lowest for HIV (4.8 %) and hypertension (4.7 %), and highest for STIs (18.0 %) and health systems (14.2 %) research.

Almost a third of the articles on hypertension in pregnancy were systematic reviews, while a relatively high proportion of interventions for malaria (21.2 %) and haemorrhage (21.8 %) were tested in trials (Table 1). Systematic reviews on HIV were uncommon (5.7 % of all HIV articles). Conversely, use of qualitative methods was more than two-fold higher in HIV research than in research on the other tracer conditions, which seldom adopted qualitative enquiry. Health systems research employed similar levels of qualitative modalities as HIV. Health systems interventions, however, were much less likely than clinical interventions to be evaluated in a trial (5.2 versus 15.8 %) and or summed in systematic reviews (6.5 versus 10.6 %).

### Comparisons across income and geographical regions (Additional file **2**: Table S1)

Low- and lower-middle income countries had a stronger focus on health systems or health promotion research than upper-middle income countries, which favoured interventional research on clinical conditions, especially on HIV and hypertension (Additional file 2: Table S1). Of note, lower-middle income countries addressed haemorrhage topics more than twice as frequently as hypertension (13.9 versus 6.3 %). Upper-middle income countries had exactly the opposite focus: 13.1 % on hypertension and 7.0 % on haemorrhage research.

**Fig. 2:**
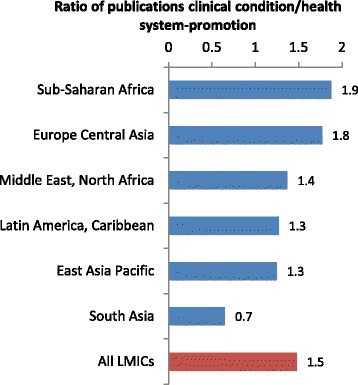
Ratio between publications on clinical conditions, and those on health systems or promotion, by region

**Fig. 3:**
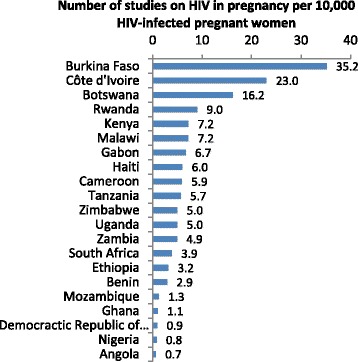
Number of studies on HIV in pregnancy per 10,000 HIV-infected pregnant women

Studies in sub-Saharan Africa made up 55.4 % of all articles. More than three quarters of research in the region (77.2 %) addressed clinical topics, mostly centred on HIV (46.9 %) and malaria (20.4 %; Additional file 2: Table S1). If studies on HIV and malaria are excluded from the analysis, sub-Saharan Africa still accounted for 37.8 % of all papers (550/1457). The proportion of research in this region on health systems and health promotion (Fig. 2), hypertension and haemorrhage is among the lowest of all the regions. South Africa accounted for 7.7 % of the 2292 studies included in the review and scientists here mostly addressed HIV research questions (61.9 %), with limited interests in health promotion (15.3 %) and haemorrhage (3.4 %). Together, Kenya and Uganda contributed almost nine percent of all studies assessed. More than half of these addressed HIV and a quarter covered malaria, while health systems research is underrepresented, and very little research addressed hypertension or haemorrhage. Systems and health promotion research was much more common in Tanzania, their East African neighbours, who produced 5.4 % of all articles (23.4 % of which were RCTs). Finally, relative to most countries in Southern Africa, HIV is over-researched in several parts of the continent (Fig. 3). Of note, for example, in Cote d’Ivoire, 93.9 % of research addressed HIV.

Latin America and the Caribbean contributed 16.7 % of all the publications studied, a large portion of which was done in Brazil (6.2 % of total studies). Half the studies in Brazil were on HIV (51.0 %), with quite low levels of health system and health promotion, or haemorrhage research. Argentina, by contrast, had a considerable focus on haemorrhage (36.4 %), while Mexican researchers covered a broader range of topics.

Studies in South Asia, which constituted only 13.7 % of studies reviewed, had a substantially greater focus on health systems or health promotion research than other regions (Fig. 2), as well as on interventions addressing maternal haemorrhage. About a quarter of studies in India included an HIV intervention (23.8 %), whilst none of the other countries in the region did so.

Most countries in East Asia Pacific clearly focused on health systems and health promotion (Fig. 2), with the notable exception of Thailand, where HIV (48.8 %) and malaria (24.4 %) research predominated. More than half the 63 studies in Europe and Central Asia were conducted in Turkey [29], which paid considerable attention to hypertension (37.8 %) and haemorrhage research (27.0 %). More than a third of the 54 studies done in the Middle East and North Africa were RCTs (37.0 %), and these mostly evaluated hypertension and haemorrhage interventions.

**Fig. 4:**
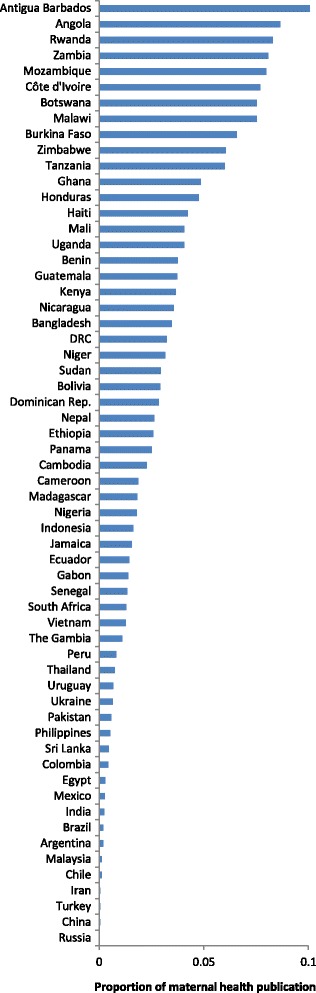
Proportion of health related publications on maternal health interventions, by country

Only five countries had more than 100 studies, namely South Africa (176), Brazil (143), India (130), Tanzania (124) and Kenya (110). Taken together, they were responsible for 29.7 % of the studies examined (683/2292). In 11 countries, maternal health interventional research made up more than five percent of all health publications (Fig. 4). By contrast, in 19 other countries these studies contributed less than one percent of the country’s research.

### Changes over time (Table **2**)

**Table 2:** Changes in publications of maternal health interventional research over time (col %)

Column %	2000–2003 (370)	2004–2007 (773)	2008–2012 (1101)	Total % (column)
Economic zone^a^				
LIC	36.9	34.4	39.5	37.3
LMIC	*29.6*	*31.1*	*34.4*	32.4
UMIC	**37.9**	**38.7**	**31.4**	35.1
Geographic zone^a^				
East Asia Pacific	*15.6*	*11*	*10.8*	11.7
Europe, Central Asia	2.9	3.9	3.1	3.3
Latin America, Caribbean	15.9	18.5	14.6	16.2
Middle East, North Africa	1.9	2.8	3.2	2.8
South Asia	12.7	12.2	15.3	13.8
Sub-Saharan Africa	54.5	55.2	56.6	55.7
Research topic^a^ (Figure 1)				
APH/PPH	9.5	8.9	8.3	8.7
Hypertension	11.1	10.3	9.7	10.2
HIV	33.5	36.5	33.8	34.7
STIs	**8.6**	**6.7**	**3.5**	5.4
Malaria	10.3	12.5	13.3	12.5
Health systems	**35.9**	**31.4**	**39.1**	35.9
Health promotion	**23.5**	**24.2**	**30.2**	27
Study design				
Systematic review	**7.8**	**8.5**	**13.7**	11
Effectiveness research	63	63.8	59.7	61.6
RCT	**17.8**	**13.2**	**10.8**	12.8
Qualitative study	**1.6**	**5.4**	**6.1**	5.1
Modelling	8.1	6.7	5.9	6.6
Mixed methods research	**1.6**	**2.3**	**3.8**	2.9
Study examines inequalities				
Inequalities examined	**6.5**	**8**	**10.4**	9
Multi-country study				
Multi-country study	5.9	7.1	7.6	7.2
Health promotion topics^a^				
Demand-side financing	**1.6**	**1.8**	**4.5**	3.1
Patient transport	1.9	1.3	2.6	2
Birth and complications preparedness	5.7	5.3	4.5	5
Community participation in maternal death audits	0.5	0.4	0.5	0.4
Maternal waiting homes	**0.8**	**0**	**1.6**	0.9
Male involvement	3.2	3.4	3.7	3.5
TBAs	4.6	4	5.4	4.8

Figures in bold *P* < 0.05. Figures in italics *P* = 0.05-0.1. Effectiveness research excludes RCTs. ^a^Multiple-response categories

**Fig. 5:**
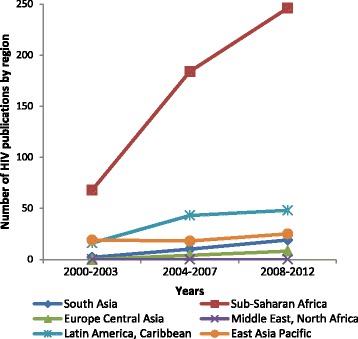
Number of publications on HIV interventional research on maternal health 2000–2012

**Fig. 6:**
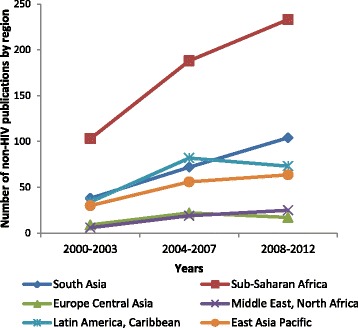
Number of publications on non-HIV interventional research on maternal health 2000–2012

The number of articles per year rose from an average of 92.5 in 2000–2003 to 193.3 in 2004–2007 and to 236.7 between 2008 and 2012. Most especially, a trend was noted towards increased research outputs in lower-middle income countries over time, making up 34.4 % of all studies by the 2008–2012 (Table 2). Upper-middle income countries conducted 31.4 % of research the third period, lower than levels in years preceding that (37–39 %). The contribution of the East Asia Pacific region to the overall body of research, however, declined over time (Figs. 5 and 6). Also, when comparing the second (2004–2007) and the third periods (2008–2012), the proportionate contribution of Latin America research declined from 18.5 to 14.6 %, matched by a rise in South Asian research, from 12.2 to 15.3 % (Figs. 5 and 6).

**Fig. 7:**
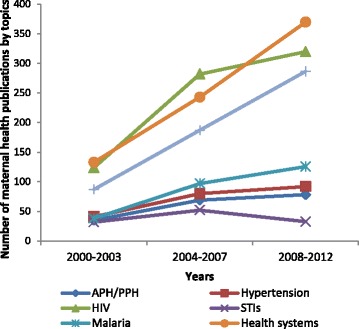
Number of publications on interventional research topics in maternal health 2000–2012

When comparing the first (2000–2003) and third (2008–2012) periods, the proportion of studies on health systems rose from 35.9 to 39.1 % and health promotion increased from 23.5 to 30.2 % (Fig. 7). STI research declined by 1.7 fold with each time period (95 % CI = 1.3–2.2 %). Merely 3.5 % of studies addressed this topic in 2008–2012, and only three quarters of these studies were based on primary data (14.8 % were modelling studies and 9.8 % systematic reviews). The number of studies on STIs, malaria, hypertension and haemorrhage were very similar in 2000–2003, but diverged markedly thereafter (Fig. 7).

The designs employed to evaluate interventions altered over time. Although the absolute numbers of studies increased progressively for each design, increments were most rapid for systematic reviews, qualitative and mixed methods research. The proportion of studies that were RCTs declined steadily over time.

The odds of a study targeting a PROGRESS-Plus group rose 1.3 fold with each time period (95 % CI = 1.1–1.6). Assessments of health financing rose over time, as did studies of maternity waiting homes, but none of the other health promotion topics examined. There was no increase in the proportion of multi-country studies.

### Impact Factor of publications in different settings and topics (Additional file **2**: Table S1)

**Fig. 8:**
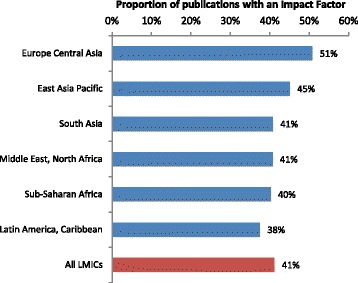
Proportion of publications in journals with an Impact Factor, by region

Forty-four percent of studies in upper-middle income countries were published in journals that have an Impact Factor, compared with 39 % of research in the other two income categories (Additional file 2: Table S1). Only 37.5 % of studies done in Latin America and Caribbean were published in journals with an Impact Factor, lower than the other regions (Fig. 8). Two thirds of malaria studies were published in journals with an Impact Factor, 8 % higher than the average of 58.8 % (Table 1). In addition to differences in the proportion of articles published in journals with an Impact Factor, patterns were noted in the magnitude of the Impact Factor across the study variables. Articles on clinical interventions were 1.4 fold more likely to be published in journals with an Impact Factor greater than 2 than were health system or health promotion articles (95 % CI OR = 1.15–1.67). HIV and malaria research also dominated high Impact Factor publications, compared to haemorrhage and hypertension studies.

A high proportion of research in China was published in journals with an Impact Factor (63.3 %), as were studies in Turkey (59.5 %). A few countries had even higher rates, such the Philippines (75 %) and Sri Lanka (80 %), but these all had fewer than 10 articles. Of the five countries with more than 100 articles, South Africa had the highest proportion of articles in Impact Factor journals (47.2 %), followed by Tanzania, India, Kenya and Brazil (all between 35 and 40 %).

### Alignment between research, and the Gross Domestic Product and burden of maternal deaths in different settings

**Fig. 9:**
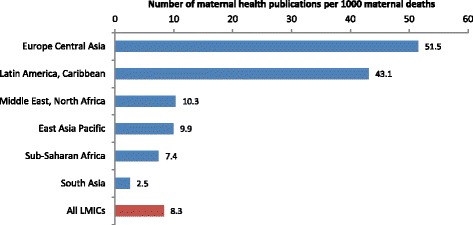
Number of maternal health publications per 1000 maternal deaths, by region

**Fig. 10:**
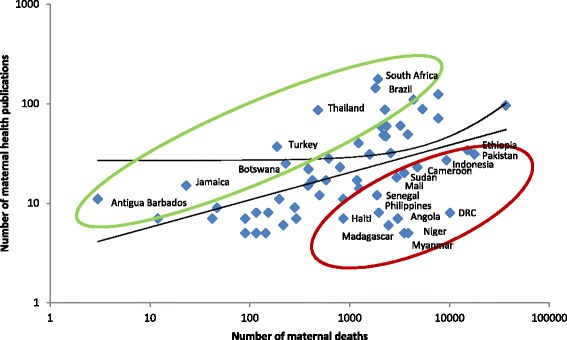
Correlation between number of maternal health publications (y axis) and number of maternal deaths (x axis). Log scale for both x and y axis and an exponential curve line fitted. Green circle shows countries with above average number of articles per deaths, red with fewer than expected

**Fig. 11:**
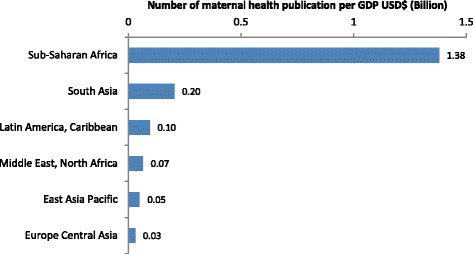
Number of maternal health publication per GDP USD$ (Billion)

**Fig. 12:**
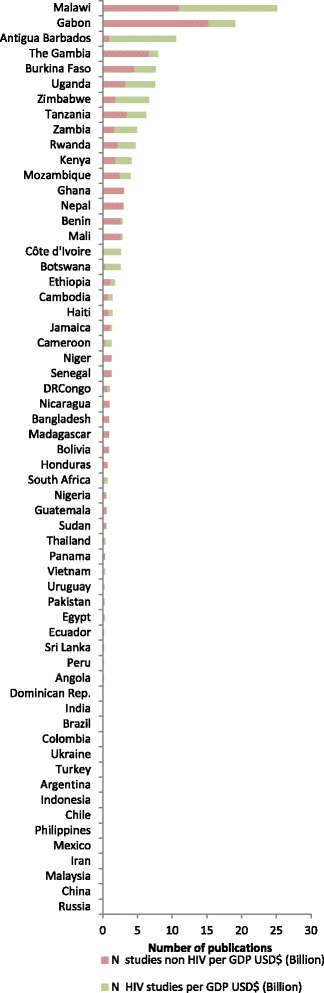
Number of publications per GDP USD$ (Billion), by country

**Fig. 13:**
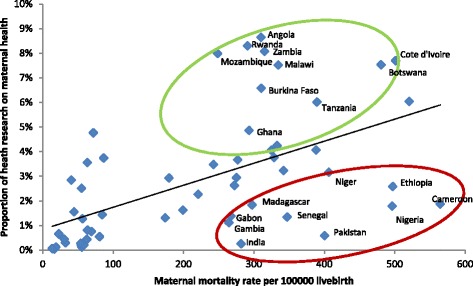
Correlation between MMR (x axis) and proportion of health research in a country on maternal health (y axis). Log scale for both x and y axis and an exponential curve line fitted. Green circle shows countries with above average attention to maternal health interventional research, red with fewer than expected

Compared to other regions, the number of publications per 1000 cases of maternal death was several-fold higher in Latin America and the Caribbean, and in Europe and Central Asia (Figs. 9 and 10). There was a marked mismatch between the research outputs, and the number of maternal deaths and GDP in South Asia (Figs. 9, 10, 11 and 12). In India in particular, there were only 0.13 publications per billion USD GDP (Fig. 12), and maternal health research formed only 0.25 % of all health publications from the country (Fig. 4). Many countries in Latin America and several large countries like China and Russia also had few papers per billion GDP and paid relatively little attention to maternal health research (Figs. 4 and 12). Conversely, a high number of countries in sub-Saharan Africa had both a high number of papers per billion GDP and a considerable proportion of their research addressed maternal health (Figs. 4 and 12). However, given the high number of maternal deaths in sub-Saharan Africa, this region had few papers per 1000 maternal deaths, second only to South Asia (Fig. 9). Finally, the maternal mortality ratio in countries and the proportion of health research on maternal health was correlated (Fig. 13). Several countries with high maternal mortality ratios, have not, however, prioritised maternal health research accordingly, such as India, Cameroon, Ethiopia, Nigeria and Pakistan.

## Discussion

Almost 18,500 articles on maternal health research in LMIC were screened, of which about a third evaluated an intervention. Marked increases occurred in research outputs over the study period, especially from 2004 onwards. Overall, around 20 % of interventional studies included a health systems or health promotion component. This proportion and that of studies on inequalities rose rapidly over time, reflecting a growing recognition of the importance of these topics. The study methods used also contributes to systematic mapping methodology, especially in the field of health inequalities. No similar studies were located in terms of size, breadth of scope and extent of international collaboration.

It has been argued before that research priorities in maternal health are not clearly aligned with the main causes of maternal mortality, or those factors responsible for inequitable health outcomes [29, 30]. The marked mismatch between the research outputs and number of maternal deaths in South Asia supports such assertions (some South Asia specific databases, like IMSEAR, were not searched, however, so figures may under-estimate actual outputs). Also, within several individual countries, it appears that negligible attention is paid to maternal health research. Prioritisation of maternal health research, redirection of research funding, addressing of research capacity gaps and the identification of interventions worth studying may all reduce the deficiencies identified. Funding and research priorities are likely to shift following adoption of the Sustainable Development Goals [31]. This provides a key opportunity for improving alignment between needs and research done.

Major causes of maternal mortality, especially haemorrhage, remain under-represented within the literature (studies per haemorrhage deaths are almost half that of hypertension) [6]. Only one in 30 publications in South Africa was on haemorrhage in pregnancy, even though it is the commonest cause of maternal mortality in the country, aside from HIV [32]. However, despite gaps in research on this topic globally, the number of deaths due to haemorrhage has declined markedly from 1990 onwards [6]. As the example of haemorrhage demonstrates, it is complex to draw conclusions from comparisons between the number of papers and burden of disease. In the haemorrhage field, perhaps the key interventions for the condition are known, and were described in literature published in the period preceding the review. Implementation scale-up might be the present priority for that field, supported by health systems evidence (a third of haemorrhage articles included health systems components), and more programmatic work, which was outside the scope of this review.

While HIV research is by some way the commonest clinical topic studied, the priority given to STI research has diminished markedly. Of note, HIV studies were considerably less likely to report maternal health outcomes than the other clinical conditions, supporting the view that these studies give relatively more attention to the health of HIV-exposed children than to HIV-infected women.

Research on health systems, even if of good quality, is less likely to be published in high-impact journals than clinical studies, possibly reflecting the relatively new journals that cover this still emerging field. Upper-middle income countries focused less on health systems or health promotion topics than the other income regions. This is concerning, as research on these topics is no less important than in other regions-large inequities, and marked failures of health care supply and demand being common to most LMICs.

Though the proportion of studies that focus on maternal health in vulnerable PROGRESS-Plus groups rose over time, this literature only constituted 10 % of publications in 2008–2012. This is a major research gap given the notable differences in the distribution of deaths and access to services within countries. For example, in most LMICs, coverage levels of skilled birth attendants are over 80 % in the richest quintile, whereas only 30 % of LMICs reported this coverage level among the poorest quintile [33]. The Sustainable Development Goals (2015–2030), in response to such deficiencies, strongly emphasize the importance of narrowing gaps in health outcomes [34]. This may spur shifts in programme and research funding priorities accordingly.

The proportion of studies employing qualitative methods and systematic review rose over time, matched by a decline in RCTs. Use of qualitative research in HIV, health systems and health promotion reflects a cognisance that context and social dimensions determine effectiveness of these interventions. Conversely, the near absence of qualitative methods in research on the other conditions might suggest an over-medicalised approach to these topics. Overall, however, the widening of research methods and of topics addressed indicates that, not only has the overall research volume increased, but also its subtlety and scope.

### Review limitations and future research

The review excluded many important topics, such as pregnancy sepsis, analyses of routine service delivery and key formative research, such as needs assessments. Many important interventions are only described in grey literature and were not included. That literature may predominately cover research on effectiveness of interventions and on interventions that address the social determinants of health, and thus the findings reported here may considerably underestimate the work done on those topics. Also, the quality of the studies was not assessed. The Impact Factor of a journal and study design provides only a limited proxy for quality. The use of country as the analytical unit ignores the fact that there are often large variations in research between provinces or regions of a country [35]. Moreover, a large coding team was necessary given the volume of work required to complete the review. This posed difficulties in standardising screening and data extraction, and articles may have been misclassified due to observer bias. This was mitigated by the use of duplicate screening and resolution of coding discordances by a more senior team member. Further, although articles were included in five languages, journals in some important languages, such as Chinese and Russian, were not. Figures presented here should thus be considered under-estimates of maternal health research in those countries in particular. Research in China has risen exponentially over time [36], though less so in Russia [37]. Lastly, research may have changed considerably since the search was done in 2012. Priorities since 2015 will likely have aligned themselves to reflect the more comprehensive multi-sectoral approach propagated in the Sustainable Develop Goals [31].

Combining the methods used here with text mining technologies in future similar studies might optimise the quality of screening and reduce the time needed for such studies [28]. In time, the study might be updated, to further examine the trajectory of patterns noted here. There may also be value in applying these methods to literature on child health, or on reproductive health more generally.

## Conclusions

The large rise in research outputs and widening of the range of research methods applied over time, suggests that the number of researchers and their skills has expanded. This allows for a more nuanced approach to addressing research questions, and, if these are aligned with research priorities, bodes well for the future of maternal health research. Changing patterns of maternal health research will require a carefully crafted research agenda reflecting knowledge needs, shifts in research funding and capacity constraints in different contexts. Declines in STI research are concerning given the burden of this disease and global efforts to eliminate mother-to-child transmission of syphilis [18]. Marked rises in research are needed in South Asia to achieve the targets set in the Sustainable Development Goals. Most importantly, perhaps, the variation in attention paid to maternal health research needs to be further interrogated and this calls for corrective action.

## Additional files

Additional file 1: Search strategy. (DOCX 25 kb) Additional file 2: Research topics and quality in countries and regions. (DOCX 38 kb)

## Abbreviations

LMICs: Low- and middle-income countries
MASCOT: Multilateral Association for Studying health inequalities and enhancing north-south and south-south COoperaTion
MH-SAR: Maternal Health and Health Systems in South Africa and Rwanda

## References

[1] WHO, UNICEF, UNFPA, World Bank Group, and the United Nations Population Division. Trends in maternal mortality: 1990 to 2015. Estimates by WHO, UNICEF, UNFPA, World Bank Group and the United Nations Population Division2015. Available from: http://www.who.int/reproductivehealth/publications/monitoring/maternal-mortality-2015/en/

[2] United Nations. The Millennium Development Goals Report 20152015. Available from: http://www.un.org/millenniumgoals/2015_MDG_Report/pdf/MDG%202015%20rev%20(July%201).pdf.

[3] United Nations Department of Economic and Social Affairs Population Division. Millennium Development Goals: 2015 Progress Chart2015. Available from: http://www.un.org/millenniumgoals/2015_MDG_Report/pdf/MDG%202015%20PC%20final.pdf

[4] Bhutta ZA, Chopra M, Axelson H, Berman P, Boerma T, Bryce J (2010). Countdown to 2015 decade report (2000–10): taking stock of maternal, newborn, and child survival. Lancet.

[5] Barros AJ, Ronsmans C, Axelson H, Loaiza E, Bertoldi AD, Franca GV (2012). Equity in maternal, newborn, and child health interventions in Countdown to 2015: a retrospective review of survey data from 54 countries. Lancet.

[6] Kassebaum NJ, Bertozzi-Villa A, Coggeshall MS, Shackelford KA, Steiner C, Heuton KR (2014). Global, regional, and national levels and causes of maternal mortality during 1990–2013: a systematic analysis for the Global Burden of Disease Study 2013. Lancet.

[7] Campbell OM, Graham WJ (2006). Strategies for reducing maternal mortality: getting on with what works. Lancet.

[8] Braine T (2005). How can health research help to save 500 000 mothers?. Bull World Health Organ.

[9] Tugwell P, Petticrew M, Kristjansson E, Welch V, Ueffing E, Waters E (2010). Assessing equity in systematic reviews: realising the recommendations of the Commission on Social Determinants of Health. BMJ.

[10] Bhutta ZA (2005). Bridging the equity gap in maternal and child health. BMJ.

[11] MASCOT/MHSAR Maternal health interventional research database. Available from: http://eppi.ioe.ac.uk/webdatabases4/Intro.aspx?ID=11.

[12] Gough D, Thomas J, Oliver S (2012). Clarifying differences between review designs and methods. Syst Rev.

[13] Oliver S, Kavanagh J, Caird J, Lorenc T, Oliver K, Harden A (2008). Health promotion, inequalities and young people’s health: a systematic review of research.

[14] O'Mara-Eves A, Brunton G, McDaid D, Oliver S, Kavanagh J, Jamal F (2013). Community engagement to reduce inequalities in health: a systematic review, meta-analysis and economic analysis.

[15] Footman K, Chersich M, Blaauw D, Campbell O, Dhana A, Kavanagh J (2014). A systematic mapping of funders of maternal health intervention research 2000 inverted question mark2012. Glob. Health.

[16] Murray CJ, Vos T, Lozano R, Naghavi M, Flaxman AD, Michaud C (2012). Disability-adjusted life years (DALYs) for 291 diseases and injuries in 21 regions, 1990–2010: a systematic analysis for the Global Burden of Disease Study 2010. Lancet.

[17] Khan KS, Wojdyla D, Say L, Gulmezoglu AM, Van Look PF (2006). WHO analysis of causes of maternal death: a systematic review. Lancet.

[18] WHO. The Global elimination of congenital syphilis : rationale and strategy for action2007. Available from: http://www.who.int/reproductivehealth/publications/rtis/9789241595858/en/.

[19] WHO. Report on global sexually transmitted infection surveillance 20132013. Available from: http://www.who.int/reproductivehealth/publications/rtis/stis-surveillance-2013/en/.

[20] The World Bank. Country and Lending Groups: Low- and middle-income economies2012. Available from: http://data.worldbank.org/about/country-classifications/country-and-lending-groups.

[21] Welch V, Petticrew M, Petkovic J, Moher D, Waters E, White H, et al. Extending the PRISMA statement to equity-focused systematic reviews (PRISMA-E 2012): explanation and elaboration. J Clin Epidemiol. 2015. 10.1016/j.jclinepi.2015.09.001 26348799

[22] WHO. Everybody business: strengthening health systems to improve health outcomes : WHO’s framework for action2007. Available from: http://www.who.int/healthsystems/strategy/everybodys_business.pdf.

[23] WHO. WHO recommendations on health promotion interventions for maternal and newborn health2015. Available from: http://www.who.int/maternal_child_adolescent/documents/health-promotion-interventions/en/. 26180864

[24] Thomson Reuters. Journal Citation Reports. 2014; Available from: http://wokinfo.com/products_tools/analytical/jcr/.

[25] Rottingen JA, Regmi S, Eide M, Young AJ, Viergever RF, Ardal C (2013). Mapping of available health research and development data: what's there, what's missing, and what role is there for a global observatory?. Lancet.

[26] UNAIDS. Global report: UNAIDS report on the global AIDS epidemic 2013. UNAIDS / JC2502/1/E2013. Available from: http://www.unaids.org/sites/default/files/media_asset/UNAIDS_Global_Report_2013_en_1.pdf.

[27] The World Bank. GDP (current$)2000-2012. Available from: http://data.worldbank.org/indicator/NY.GDP.MKTP.CD?page=2.

[28] Shemilt I, Simon A, Hollands GJ, Marteau TM, Ogilvie D, O'Mara-Eves A (2014). Pinpointing needles in giant haystacks: use of text mining to reduce impractical screening workload in extremely large scoping reviews. Res Synth Methods.

[29] Vargas E, Becerril-Montekio V, Gonzalez-Block MA, Akweongo P, Hazel CN, Cuembelo Mde F (2016). Mapping the use of research to support strategies tackling maternal and child health inequities: evidence from six countries in Africa and Latin America. Health Res Policy Syst.

[30] Gil-Gonzalez D, Carrasco-Portino M, Ruiz MT (2006). Knowledge gaps in scientific literature on maternal mortality: a systematic review. Bull World Health Organ.

[31] Bhutta ZA, Chopra M (2016). Moving ahead: what will a renewed Countdown to 2030 for Women and Children look like?. Lancet.

[32] Moodley J, Pattinson RC, Fawcus S, Schoon MG, Moran N, Shweni PM (2014). The confidential enquiry into maternal deaths in South Africa: a case study. Bjog.

[33] WHO. State of inequality: reproductive, maternal, newborn and child health2015. Available from: http://apps.who.int/iris/handle/10665/164590.

[34] United Nations (UN) Department of Economic and Social Affairs Population Division. Transforming our world: The 2030 agenda for sustainable development2015. Available from: https://sustainabledevelopment.un.org/post2015/transformingourworld/publication.

[35] Montgomery AL, Ram U, Kumar R, Jha P (2014). Maternal mortality in India: causes and healthcare service use based on a nationally representative survey. PLoS One.

[36] Medline (Pubmed). Number of Medline hits on China 2016. Available from: http://www.ncbi.nlm.nih.gov/pubmed/?term=china*.

[37] Medline (Pubmed). Number of Medline hits on Russia 2016. Available from: http://www.ncbi.nlm.nih.gov/pubmed/?term=russia*.

