# Supplementary material for: Mapping of research on maternal health interventions in low- and middle-income countries: a review of 2292 publications between 2000 and 2012
Source: Global Health. 2016 Sep 6;12(1):52. doi: 10.1186/s12992-016-0189-1 (PMC5011860; doi:10.1186/s12992-016-0189-1)
Supplement: Additional file 2: — Research topics and quality in countries and regions. (DOCX 38 kb) [file 12992_2016_189_MOESM2_ESM.docx]

**Additional file 2: Table S1 Research topics and quality in countries and regions**

|  | **RCT design** | **Has Impact Factor** | **Health systems** | **Health promotion** | **APH/PPH** | **Hypertension** | **HIV** | **STIs** | **Malaria** | **Total N** | **Total % (column)** |
| --- | --- | --- | --- | --- | --- | --- | --- | --- | --- | --- | --- |
| **Total % (row)** | **13.1%** | **41.2%** | **36.2%** | **27.3%** | **8.8%** | **10.1%** | **34.6%** | **5.3%** | **12.3%** | 2292 |  |
| **Economic region** |  |  |  |  |  |  |  |  |  |  |  |
| LIC | **18.2** | 39.5 | **42** | **34.1** | **5.2** | **3.9** | **39.9** | *3.9* | **18.9** | 724 | 37.2% |
| LMIC | 13.9 | 39.7 | 38.5 | 27.4 | **13.9** | *6.3* | **27.6** | 5.1 | 14.6 | 624 | 32.1% |
| UMIC | 15.3 | **43.9** | **30.4** | **20.2** | *7* | **13.1** | **42** | **6.7** | **3.9** | 688 | 35.4% |
| **Geographical region and countries** |  |  |  |  |  |  |  |  |  |  |  |
| **East Asia Pacific** |  |  |  |  |  |  |  |  |  |  |  |
| **Region total** | 17.3 | 45.1 | 40.3 | 28.3 | *11.5* | 4.9 | 27.9 | 8.4 | 10.2 | 226 | 11.6% |
| Cambodia | 0 | 36.4 | **72.7** | **63.6** | 9.1 | 9.1 | 45.5 | 9.1 | 0 | 11 | 0.5% |
| China | 18.3 | **63.3** | 43.3 | *36.7* | 13.3 | *3.3* | **11.7** | **20** | **0** | 60 | 2.6% |
| Indonesia | 3.7 | 40.7 | **77.8** | 40.7 | 11.1 | *0* | *3.7* | 3.7 | 3.7 | 27 | 1.2% |
| Malaysia | 28.6 | 71.4 | 57.1 | 28.6 | *28.6* | **42.9** | *0* | 0 | 0 | 7 | 0.3% |
| Myanmar | 0 | 40 | 60 | 60 | 20 | 0 | 0 | 0 | 20 | 5 | 0.2% |
| Thailand | **23.3** | 38.4 | **15.1** | **14** | 7 | *4.7* | **48.8** | 4.7 | **24.4** | 86 | 3.8% |
| Philippines | 25 | *75* | **87.5** | 12.5 | 0 | 0 | **0** | 12.5 | 0 | 8 | 0.3% |
| Vietnam | *26.1* | 39.1 | 21.7 | 13 | **39.1** | 0 | 39.1 | 0 | *0* | 23 | 1.0% |
| **Europe Central Asia** |  |  |  |  |  |  |  |  |  |  |  |
| **Region total** | 14.3 | *50.8* | 28.6 | 20.6 | **15.9** | **23.8** | **19** | 6.3 | **0** | 63 | 3.2% |
| Russian Federation | 0 | 28.6 | 28.6 | 14.3 | 0 | 0 | 42.9 | 14.3 | 0 | 7 | 0.3% |
| Turkey | 21.6 | **59.5** | *21.6* | 24.3 | **27** | **37.8** | **2.7** | 2.7 | **0** | 37 | 1.6% |
| Ukraine | 0 | 62.5 | 37.5 | 12.5 | 0 | 0 | **75** | 12.5 | 0 | 8 | 0.3% |
| **Latin America, Caribbean** |  |  |  |  |  |  |  |  |  |  |  |
| **Region total** | 10.2 | 37.5 | 37.8 | 27.1 | 8 | 13.5 | 33.8 | 8 | 0.6 | 325 | 16.7% |
| Antigua Barbados | 9.1 | 36.4 | 18.2 | 9.1 | 0 | 9.1 | **90.9** | 0 | 0 | 11 | 0.5% |
| Argentina | **27.3** | 40.9 | 22.7 | 18.2 | **36.4** | 4.5 | 36.4 | 9.1 | *0* | 22 | 1.0% |
| Bolivia | 0 | 50 | 41.7 | *50* | 0 | 0 | **0** | **66.7** | 0 | 12 | 0.5% |
| Colombia | 17.6 | 29.4 | 35.3 | 23.5 | 17.6 | **41.2** | **11.8** | 0 | 0 | 17 | 0.7% |
| Dominican Republic | 0 | 40 | 40 | 20 | 20 | 0 | 60 | 0 | 0 | 5 | 0.2% |
| Ecuador | 22.2 | 66.7 | 44.4 | 11.1 | **33.3** | 22.2 | **0** | 11.1 | 0 | 9 | 0.4% |
| Chile | 22.2 | 33.3 | 33.3 | 33.3 | 0 | 11.1 | 33.3 | 11.1 | 0 | 9 | 0.4% |
| Brazil | **6.3** | 35.7 | *28.7* | **17.5** | **0** | 10.5 | **51** | 4.9 | **1.4** | 143 | 6.2% |
| Guatemala | 11.8 | 47.1 | **82.4** | **64.7** | 11.8 | 0 | **0** | 0 | 0 | 17 | 0.7% |
| Haiti | 0 | 28.6 | 42.9 | 28.6 | 0 | 0 | 42.9 | **42.9** | 0 | 7 | 0.3% |
| Honduras | 25 | 50 | **75** | 25 | 12.5 | 0 | **0** | 0 | 0 | 8 | 0.3% |
| Jamaica | 20 | 46.7 | 46.7 | 33.3 | 20 | **26.7** | 20 | 13.3 | 0 | 15 | 0.7% |
| Mexico | 10 | 35 | 35 | 37.5 | 10 | 15 | 30 | 0 | **0** | 40 | 1.7% |
| Nicaragua | 0 | 14.3 | **85.7** | 42.9 | 0 | 0 | *0* | 0 | 0 | 7 | 0.3% |
| Panama | 28.6 | 28.6 | **0** | 14.3 | 0 | **100** | *0* | 0 | 0 | 7 | 0.3% |
| Peru | 6.7 | 53.3 | **73.3** | 20 | 0 | 6.7 | 33.3 | **20** | 0 | 15 | 0.7% |
| Uruguay | 0 | 42.9 | 42.9 | 14.3 | *28.6* | 0 | 28.6 | 0 | 0 | 7 | 0.3% |
| **Middle East, North Africa** |  |  |  |  |  |  |  |  |  |  |  |
| **Region total** | **37** | 40.7 | 35.2 | 29.6 | **31.5** | **22.2** | **0** | 5.6 | **0** | 54 | 2.8% |
| Iran Islamic Republic | **63.6** | 36.4 | 27.3 | 27.3 | 9.1 | **45.5** | **0** | 9.1 | 0 | 11 | 0.5% |
| Egypt | **35.7** | 50 | **17.9** | 25 | **53.6** | 17.9 | **0** | 3.6 | **0** | 28 | 1.2% |
| **South Asia** |  |  |  |  |  |  |  |  |  |  |  |
| **Region total** | 16.9 | 40.8 | **53.2** | **47.9** | **15.4** | **12** | **12** | 3.4 | **2.6** | 267 | 13.7% |
| Bangladesh | 12.7 | 39.4 | **74.6** | **64.8** | 9.9 | **18.3** | **0** | **0** | **0** | 71 | 3.1% |
| India | 16.9 | 37.7 | 32.3 | **35.4** | **17.7** | 13.1 | **23.8** | 6.2 | **4.6** | 130 | 5.7% |
| Nepal | 16.1 | 38.7 | **74.2** | **67.7** | 12.9 | *0* | **0** | 0 | **0** | 31 | 1.4% |
| Pakistan | **29** | 41.9 | **61.3** | **54.8** | 16.1 | 3.2 | **3.2** | 3.2 | **0** | 31 | 1.4% |
| Sri Lanka | 0 | *80* | 40 | 0 | 20 | 20 | 0 | 0 | 20 | 5 | 0.2% |
| **Sub-Saharan Africa** |  |  |  |  |  |  |  |  |  |  |  |
| **Region total** | 16.5 | 40.2 | **32.6** | **22.9** | **5.9** | **4.4** | **46.9** | **3.9** | **20.4** | 1080 | 55.5% |
| Angola | 0 | 28.6 | 42.9 | 14.3 | **42.9** | 0 | 14.3 | 0 | 0 | 7 | 0.3% |
| Benin | **35.7** | 28.6 | 28.6 | 28.6 | 7.1 | 7.1 | **7.1** | 0 | **57.1** | 14 | 0.6% |
| Botswana | **28** | 44 | **12** | **8** | 0 | *0* | **84** | **16** | *0* | 25 | 1.1% |
| Burkina Faso | **29.2** | 39.6 | 37.5 | 33.3 | *2.1* | *2.1* | 39.6 | 2.1 | **27.1** | 48 | 2.1% |
| Cameroon | 4.3 | *60.9* | 26.1 | 13 | 4.3 | 0 | **69.6** | 0 | 17.4 | 23 | 1.0% |
| Democractic Republic of Congo | 0 | *12.5* | 37.5 | 12.5 | 0 | 0 | 37.5 | 12.5 | **62.5** | 8 | 0.3% |
| Côte d'Ivoire | 16.3 | 32.7 | **6.1** | **6.1** | *2* | **0** | **93.9** | 4.1 | **2** | 49 | 2.1% |
| Ethiopia | 5.9 | 38.2 | *50* | **44.1** | 11.8 | 2.9 | 35.3 | 0 | 11.8 | 34 | 1.5% |
| Gabon | 0 | 60 | *0* | 0 | 0 | 0 | 20 | 0 | **80** | 5 | 0.2% |
| The Gambia | **66.7** | 66.7 | 33.3 | 33.3 | **33.3** | 0 | 16.7 | 0 | **50** | 6 | 0.3% |
| Ghana | 18.6 | 37.3 | **55.9** | 27.1 | **20.3** | 5.1 | **1.7** | 1.7 | **32.2** | 59 | 2.6% |
| Madagascar | 0 | 50 | 50 | 50 | 16.7 | 0 | *0* | 0 | **50** | 6 | 0.3% |
| Malawi | 16.1 | 39.1 | 34.5 | 21.8 | 4.6 | **2.3** | **56.3** | 3.4 | *18.4* | 87 | 3.8% |
| Mali | 11.1 | 44.4 | **61.1** | **50** | 16.7 | 5.6 | **5.6** | 5.6 | **33.3** | 18 | 0.8% |
| Mozambique | 12.5 | 34.4 | 40.6 | 15.6 | 3.1 | 3.1 | 37.5 | *12.5* | 18.8 | 32 | 1.4% |
| Niger | 20 | 40 | 60 | 60 | 0 | 0 | 0 | 0 | 20 | 5 | 0.2% |
| Rwanda | 5.9 | 47.1 | 41.2 | 17.6 | 0 | 0 | 52.9 | 0 | 17.6 | 17 | 0.7% |
| Senegal | 0 | 33.3 | **75** | *50* | 16.7 | 8.3 | **0** | 0 | 16.7 | 12 | 0.5% |
| Sudan | 15 | 25 | **15** | 15 | 0 | 0 | *15* | 5 | **75** | 20 | 0.9% |
| Uganda | **22.7** | 48.9 | 28.4 | 25 | **2.3** | **2.3** | **56.8** | 4.5 | **21.6** | 88 | 3.8% |
| Zambia | 13.6 | *52.5* | 32.2 | *16.9* | *1.7* | **0** | **66.1** | 3.4 | 13.6 | 59 | 2.6% |
| Kenya | 18.2 | 37.3 | **27.3** | 21.8 | **0.9** | **0.9** | **56.4** | 6.4 | **27.3** | 110 | 4.8% |
| Nigeria | 6.3 | 39.6 | **24** | **16.7** | **16.7** | 12.5 | **16.7** | 2.1 | **37.5** | 96 | 4.2% |
| South Africa | 15.9 | *47.2* | 31.8 | **15.3** | **3.4** | 10.2 | **61.9** | 5.1 | **0** | 176 | 7.7% |
| Tanzania | **23.4** | 38.7 | 41.1 | 28.2 | *4* | **3.2** | **44.4** | 4 | **22.6** | 124 | 5.4% |
| Zimbabwe | 19.1 | 36.2 | 25.5 | 29.8 | 2.1 | 6.4 | **72.3** | *0* | 6.4 | 47 | 2.1% |

Row % i.e. the proportion of studies in a region/country with the characteristic named in the column header. Figures in bold P<0.05. Figures in italics P=0.05-0.1 *Multiple-response categories. Only countries with ≥5 papers were included
